# The Effects of Pectin–Honey Hydrogel in a Contaminated Chronic Hernia Model in Rats

**DOI:** 10.3390/gels9100811

**Published:** 2023-10-11

**Authors:** Anna Cerullo, Gessica Giusto, Lorella Maniscalco, Patrizia Nebbia, Mitzy Mauthe von Degerfeld, Matteo Serpieri, Cristina Vercelli, Marco Gandini

**Affiliations:** Department of Veterinary Sciences, University of Turin, Largo Paolo Braccini, 5, Grugliasco, 10095 Turin, Italy; anna.cerullo@unito.it (A.C.); gessica.giusto@unito.it (G.G.); lorella.maniscalco@unito.it (L.M.); patrizia.nebbia@unito.it (P.N.); mitzy.mauthe@unito.it (M.M.v.D.); matteo.serpieri@unito.it (M.S.); cristina.vercelli@unito.it (C.V.)

**Keywords:** adhesion, bacterial contamination, hernia healing, pectin–honey-hydrogel, polypropylene mesh, rats

## Abstract

Incisional hernia is a frequent complication after abdominal surgery. A previous study on rats evaluated the use of a Pectin–Honey Hydrogel (PHH)-coated polypropylene (PP) mesh for the healing of acute hernias. However, there are no studies investigating the use of PHH in association with PP mesh in chronic contaminated hernia. The aims of this study are to assess the effectiveness of PHH in promoting abdominal hernia repaired with PP mesh and in counteracting infection. Twenty Sprague Dawley male rats were enrolled and a full thickness defect was made in the abdominal wall. The defect was repaired after 28 days using a PP mesh, and a culture medium (Tryptone Soy Broth, Oxoid) was spread onto the mesh to contaminate wounds in both groups. The rats were randomly assigned to a treated or untreated group. In the treated group, a PHH was applied on the mesh before skin closure. At euthanasia—14 days after surgery—macroscopical, microbiological and histopathological evaluations were performed, with a score attributed for signs of inflammation. An immunohistochemical investigation against COX-2 was also performed. Adhesions were more severe (*p* = 0.0014) and extended (*p* = 0.0021) in the untreated group. Bacteriological results were not significantly different between groups. Both groups showed moderate to severe values (score > 2) in terms of reparative and inflammatory reactions at histopathological levels. The use of PHH in association with PP mesh could reduce adhesion formation, extension and severity compared to PP mesh alone. No differences in terms of wound healing, contamination and grade of inflammation were reported between groups.

## 1. Introduction

Abdominal hernia is a common complication after surgery [1], especially following complications in the wound-healing phase [2]. Abdominal hernia, if untreated, can cause further problems, such as small intestinal strangulation [3], and is associated with impaired quality of life and increased healthcare costs [4,5]. Although several repair techniques have been proposed, polypropylene (PP) mesh application has ultimately been the most effective technique [3,6]. PP mesh has the advantage of being flexible, chemically inert, stable, nonimmunogenic and non-toxic, with high tensile strength and low susceptibility to infections [3,7,8]. However, the possibility of secondary infections following the application of a foreign body, even if biocompatible, is not to be excluded [9]. The risk of infection is high when the level of contamination exceeds 10^5^ bacteria per gram of tissue [10], although lower doses (10^2^ organisms per gram of tissue) may be required if foreign material, such as prostheses, meshes or sutures, is present. [11]. The use of foreign materials could promote the growing of bacteria, which can develop a series of mechanisms that make them resistant, such as bacterial adherence and biofilm formation on mesh surfaces [12,13]. Therefore, it is important to adopt preventive measures to control implant infection, since the bacteria involved are often multi-resistant and, therefore, conventional therapies can be ineffective [14].

Several biomaterials were studied and tested to evaluate how to repair hernias and limit side effects such as immune system overreaction and adhesions, as well as reduce the risk of bacterial infection. For this reason, several membranes of animal origin [15,16,17], or synthetic meshes associated with antibacterial and/or anti-inflammatory components such as nitric oxide-releasing silica nanoparticles [18], meshes coated with antimicrobial metals (i.e., Ag, Zn) and mesh coated/impregnated with a combination of different antimicrobials [19] have been developed. Furthermore, the increase in antibiotic-resistant microorganisms has led to a revaluation of the therapeutic use of ancient remedies, such as honey. Previous studies have evaluated the in vitro and in vivo efficacy of Pectin–Honey Hydrogels (PHH) [20,21,22]. Honey is a component used since ancient times for its antibacterial and anti-inflammatory characteristics. Pectin has been widely used as a scaffold for wound dressing in tissue engineering to improve wound healing, and it is biocompatible, biodegradable and nontoxic [20]. PHHs are equally effective in promoting wound healing and reducing the onset of peritoneal adhesions [23]. A previous study also evaluated the use of polypropylene mesh coated with PHH to help heal acute hernias [24].

Polypropylene mesh is the most used material and has proven to have several benefits [19]. Nevertheless, this material induces an acute and intense inflammatory reaction that may lead to adhesion formation, mesh extrusion, contraction, fistulation and chronic abdominal pain [25], sometimes resulting in seroma and secondary infections with a necessity of debridement or mesh removal [26]. A PHH–PP mesh could limit the risks of bacterial infection and antimicrobial resistance, reducing the risk of postoperative adhesions. Furthermore, mesh reinforcement trials of ventral hernia repair were mostly performed in a clean field, while the safety of mesh in contaminated ventral hernia repair has only been evaluated in few studies [27,28,29,30]. It is important to consider that, while its application in human surgery may have limited side effects due to the patient’s controlled postoperative management, in veterinary medicine, there is a greater possibility of wound contamination via contact with bacteria present on the animal itself and in the hospitalization area. However, few studies test the application of mesh in a contaminated environment [29,31]. Repairing an abdominal hernia in a contaminated field can be challenging due to the increased risk of infection and complications. Nevertheless, surgeons may still need to perform hernia repairs in contaminated fields (i.e., when the risk of delaying surgery is deemed higher than the risk of infection). The use of PPH-coated meshes has been tested for the repair of acute abdominal hernias, but there are no studies concerning the effects of the use of PHH–PP meshes to repair abdominal hernias in a contaminated environment. Wound healing is a complex process involving the migration, proliferation, interaction and differentiation of different cell types. The presence of bacteria during hernia healing could alter or strongly slow the repair process. The use of mesh for abdominal hernia repair increases the risk of infection during the healing process because, in the presence of foreign bodies, the bacteria amount sufficient to spread infection is reduced. The use of PHH could represent a valid strategy to counteract bacterial growth without altering the normal hernia healing mechanism.

The aim of this study was to assess if the use of PHH before skin closure improves the healing of abdominal hernias repaired with PP mesh in a contaminated field in terms of adhesion formation, incisional infections, tissue healing and grade of inflammation. The hypothesis of this study is that the use of a PHH–PP mesh would improve fascial healing after hernia repair in a contaminated field, reducing the risk of abdominal adhesions and surgical site infection. Macroscopic, microbiological, histological and immunohistochemistry assessments were performed to compare the type of healing of wounds treated with PHH–PP meshes with those in which the hernia was repaired as per routine practice with only the use of a PP mesh.

## 2. Results

Among the 20 rats used for the experiment, 3 specimens died between postoperative day 1 and day 2. This mortality rate is considered normal for rats of this age in experimental studies.

### 2.1. Macroscopical Evaluation

**Figure 1:**
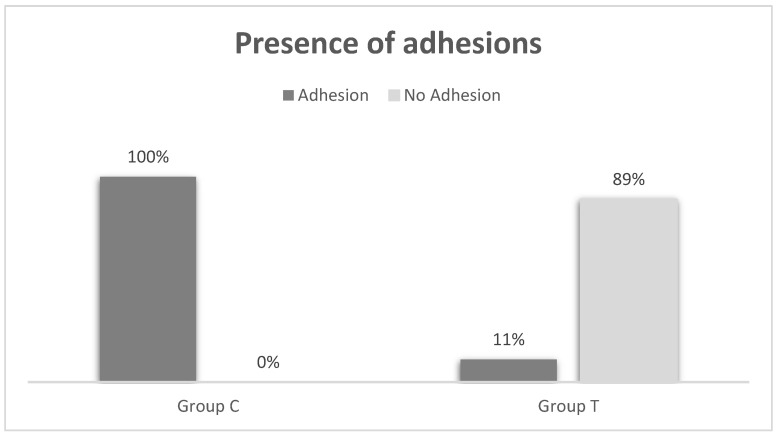
Details related to the percentage of adhesions reported in Group T and in Group C.

In the remaining 17 rats, the macroscopic evaluation of the wounds revealed a higher incidence of adhesions in Group C (8/8, 100%) compared to Group T (1/9, 11%; *p* = 0.0004). In 3 out of 8 cases of Group C, there was a persistence of the hernia defect, while in 2 out of 9 cases of Group T, the defect involved the skin and subcutaneous tissue, but the linea alba remained normal. In 7 cases of Group C, adhesions were observed between omentum and linea alba, while in 1 case, the adhesion was between the linea alba and the bladder. Details regarding the distribution of adhesions between groups are depicted in Figure 1.

**Figure 2:**
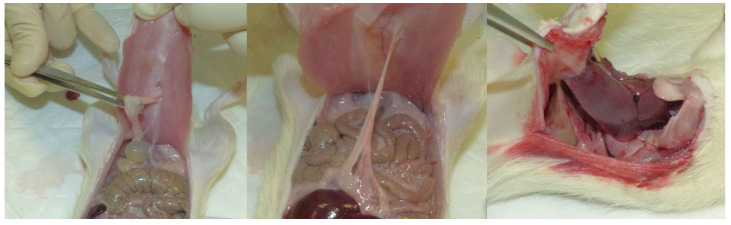
Presence of adhesions between linea alba and organs in three rats from Group C. The first figure on the left depicts the adhesion between the linea alba and the bladder. In the middle figure, the adhesion between the linea alba and intestine is reported, while the last figure shows the adhesion between the abdominal wall and the liver.

The median score (range) for adhesion severity was 0 (0–3) in Group T and 2 (1–3) in Group C, indicating a higher severity score in Group C (*p* = 0.0014). The median extension area (range) of adhesions was 0 (0–4) in Group T and 1 (1–2) in Group C, demonstrating a significantly larger area of adhesions in Group C (*p* = 0.0021; Figure 2). There were no abscesses on the wounds or mesh contractures in either group. The presence of skin erythema between the two groups was not significant (*p* = 1), with only one case reported in each group (score 1).

### 2.2. Bacteriological Evaluation

The median number (range) of viable cells per swab (CFU/swab) after 24 h was 2 (0–25) in Group T and 0 (0–100) in Group C, resulting in no significant difference between the two groups (*p* = 0.672). After 48 h, the median number (range) of CFU/swab was 1 (0–28) in Group T and 1.5 (0–406) in Group C, with no significant differences between the two groups (*p* = 0.869). However, the number of CFU/mL tended to be higher in Group C compared to Group T. Further details on CFU after 24 h and 48 h are provided in Table 1.

### 2.3. Histological Evaluation and Immunohistochemical Analysis

**Figure 3:**
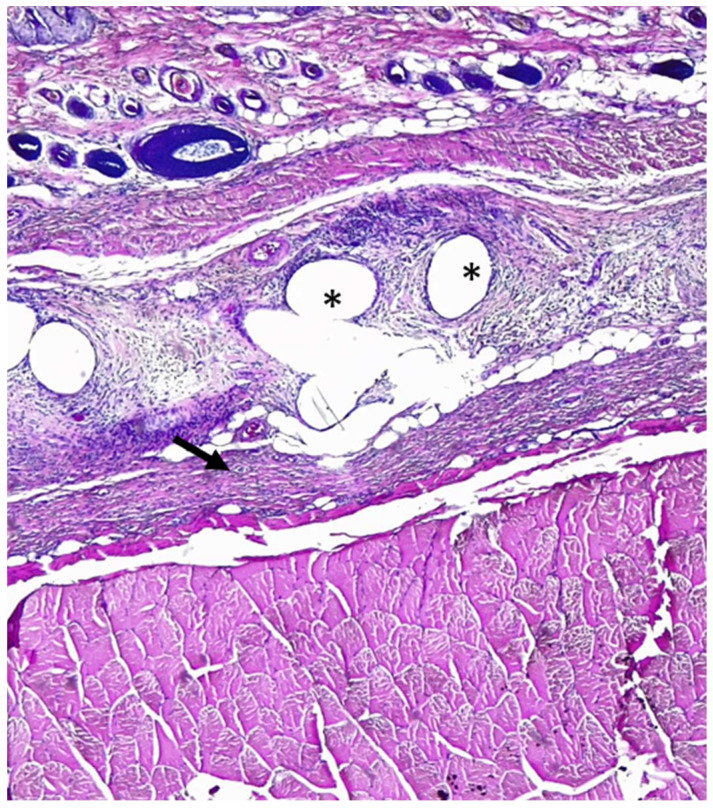
Rat abdominal wall tissues histological section. Multifocal granulomas (asterisk) are present (inflammatory response score 2) and thin layer of fibrosis (arrow-tissue maturation score 2). Hematoxylin–eosin staining; 40× magnification.

**Figure 4:**
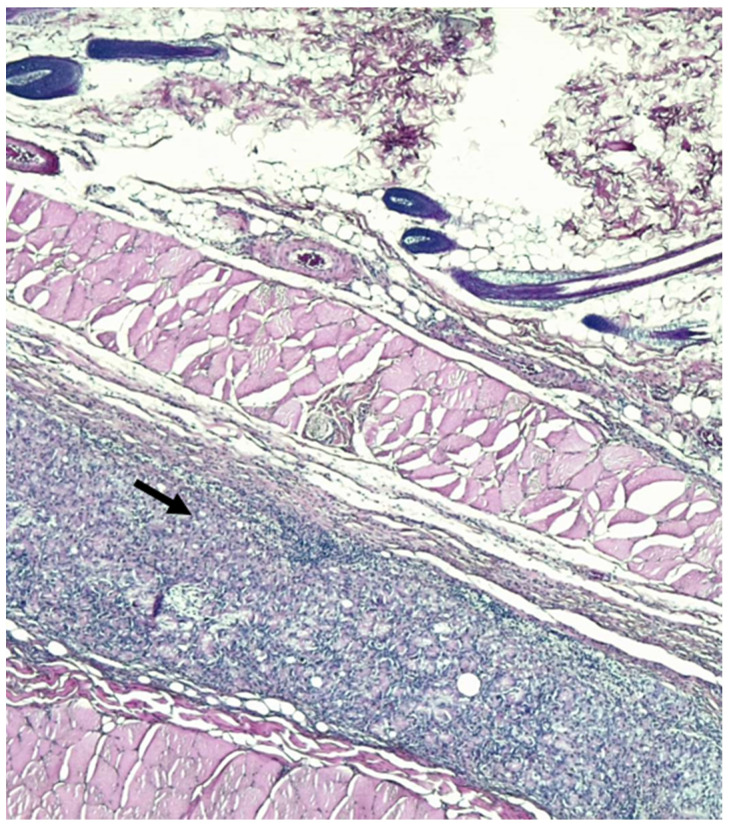
Rat abdominal wall tissue histological sections. Arrow indicates dense granular tissue with fibroblasts and inflammatory infiltrates (score 3 of inflammatory reaction in host tissue). Hematoxylin–eosin staining; 40× magnification.

**Figure 5:**
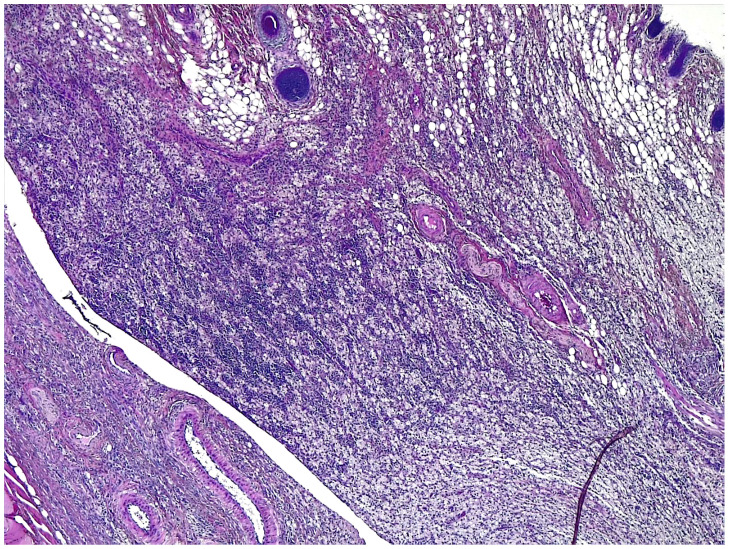
Rat abdominal wall tissue histological sections. Mass of inflammatory cells without permeating connective tissue in host tissue (score 4). Hematoxylin–eosin staining; 40× magnification.

Histological examination revealed that in both the T and C groups, nearly all subjects exhibited moderate to severe values (score > 2) in terms of reparative and inflammatory reactions (Figure 3, Figure 4 and Figure 5), except for subject C5, who had lower tissue maturation. The observed inflammatory infiltrate consisted of aggregates of macrophages (granulomatous inflammation) or a mixed inflammatory infiltrate primarily composed of lymphocytes, plasma cells, and macrophages, with fewer neutrophils.

These results are summarized in Table 2, which demonstrates the absence of any statistical difference between the groups.

**Figure 6:**
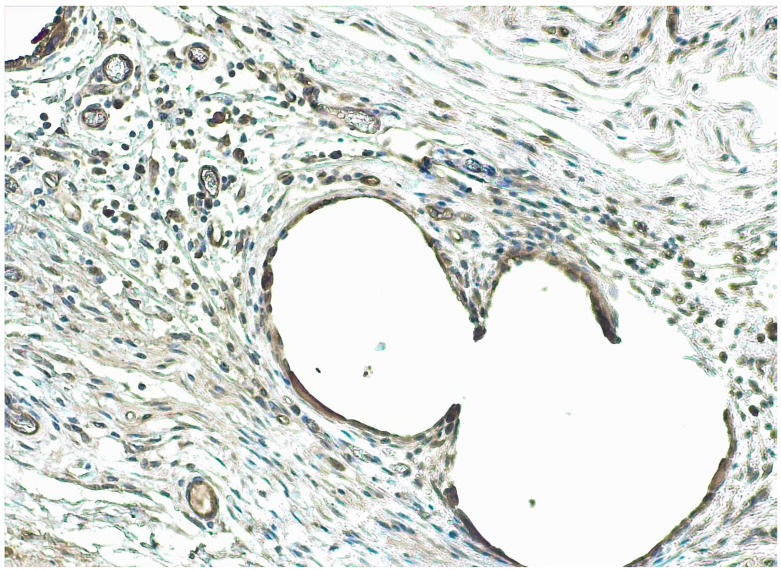
Abdominal wall tissue immunohistochemical staining against COX-2. a: 25–50% of COX-2 immunopositive inflammatory cells (score 2) with a moderate intensity (score 2), 400× magnification; b: 51–100% of COX-2 immunopositive cells (score 3) with a strong intensity (score 3), 200× magnification. DAB chromogen, hematoxylin counterstain.

**Figure 7:**
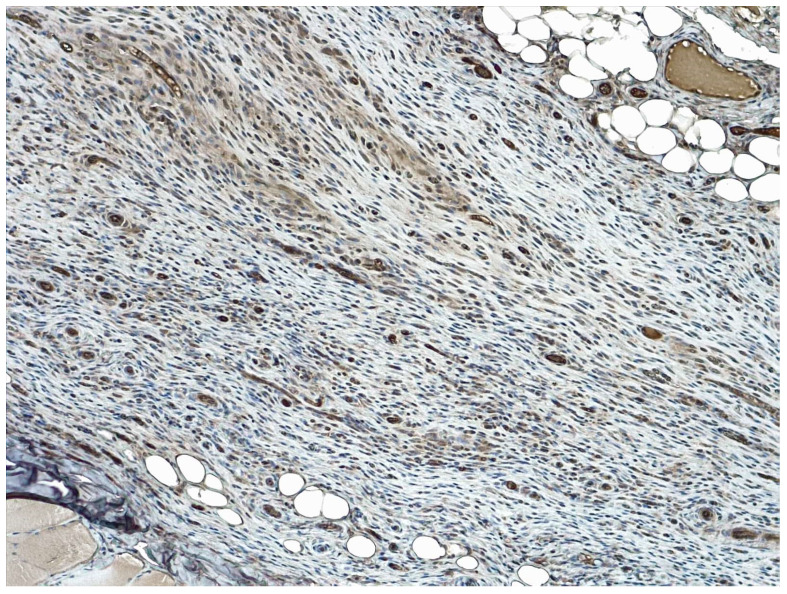
Abdominal wall tissue immunohistochemical staining against COX-2. There are 51–100% of COX-2 immunopositive inflammatory cells (score 3) with a strong intensity (score 3), 200× magnification. DAB chromogen, hematoxylin counterstain.

Immunopositivity for COX-2 was observed in inflammatory cells and multifocally in reactive fibroblasts or endothelium. Following the approach of Pereira-Lucena and colleagues (2014), *n* of positive inflammatory cells was considered to calculate the score. The median score for the percentage of positive cells was 2 (1–3) in Group T and 2 (1–3) in Group C, indicating no significant differences between the groups (*p* = 0.59). Similarly, the median score for COX-2 intensity was 2 (1–3) in both Group T and Group C (*p* = 0.52). Details regarding the COX-2 expression score in the treated and untreated groups are reported in Table 3 and are shown in Figure 6 and Figure 7.

## 3. Discussion

The application of PHH as an apposition on PP mesh prevents the onset of abdominal adhesions, while bacterial contamination is not limited by its use, as no differences were found between the two groups. This condition may be related to the difficulty of protecting wounds from lambing, as animals have little tolerance for the use of Elizabethan collars or bandages. Additionally, the presence of honey may have further encouraged the animals to lick their wounds. Regarding macroscopic evaluations, the results of this study showed no differences between the two groups except for the presence, extension and severity of adhesions. Furthermore, histological evaluation of the slides stained with H&E confirmed the absence of infections, and no differences in terms of inflammation between two groups. The use of synthetic mesh for repairing abdominal hernias, which remains in contact with the viscera, can routinely induce the onset of adhesions [32]. Several new devices have been compared to the most-used PP mesh, such as bioabsorbable membranes composed of sodium hyaluronate and chemically derivatized carboxymethylcellulose [32,33], or titanium-coated filament PP or omega-3 fatty acid-coated filament PP [34]. Previous studies have also reported a higher prevalence of peritoneal adhesions following the use of PP mesh compared to other types of mesh [32,33,35,36] or PP mesh combined with other components [34,36]. Nevertheless, other authors report the use of PP mesh as effective and safe, with acceptable morbidity, good results and only a few side effects [6,37]. At the same time, Giusto and colleagues (2016) reported that the insertion of PHH into the abdomen reduces the occurrence of peritoneal adhesions [23]. Based on these findings, the present study aimed to evaluate the use of PHH in combination with PP mesh for repairing abdominal hernias in a contaminated environment. The results of this study confirm the effectiveness of using PHH in conjunction with PP mesh to reduce the occurrence of abdominal adhesions. A previous study reported no significant difference in the occurrence of abdominal adhesions when comparing the use of PP mesh to PHH-coated mesh [24]. However, it is worth noting that the study by Vercelli et al. (2021) was conducted on an acute hernia model, which may present different healing conditions to those observed in our study. Moreover, it remains unclear whether adhesions were caused by the suture material used to secure the meshes rather than the meshes themselves [24].

While the use of PHH has not demonstrated significantly greater efficacy in terms of antibacterial and anti-inflammatory action compared to the untreated group, the potential of these devices to reduce the occurrence of abdominal adhesions justifies their application in clinical settings. Combining PHH with PP mesh offers the advantages of utilizing PP meshes, in terms of flexibility and strength, while mitigating their adverse effects such as adhesion formation. Although some studies have shown that PP mesh may be less effective than other devices, it remains the most commonly used option [6] due to its strength, resistance and the ability to facilitate fibroblasts’ penetration, ensuring proper mesh incorporation [24], all while maintaining cost-effectiveness.

One limitation of the study is the reduced number of animals used, which is based on ministerial regulations aimed at safeguarding animal welfare. Additionally, the surgical technique resulted in a high mortality rate among subjects, further restricting the available pool of animals. The relatively low incidence of signs of infection in the control group, despite the experimental contamination of wounds, may be attributed to both the low pathogenicity of the bacterial broth used and the robust immune response of the rats employed. The presence of an infection could have led to different outcomes.

## 4. Conclusions

This study showed that the use of PHH yielded excellent results in peritoneal regeneration, effectively preventing the development of peritoneal adhesions after hernia repair with PP meshes in a contaminated surgical field. PHH promotes rapid tissue regeneration within the mesh, underscoring the positive impact of honey and pectin in the hernia healing process. Future research endeavors should investigate the effectiveness of these devices in countering infections caused by multi-resistant bacteria and assess the applicability of PHH with various types of mesh.

## 5. Materials and Methods

### 5.1. Animals

All procedures received approval from both the Bioethical Committee of the University of Turin and the Italian Ministry of Health (Protocol Number 635/2022-PR, 13 October 2022). The study involved a total of twenty adult male Sprague-Dawley rats, with a weight ranging from 250 to 275 g, procured from Envigo Rms S.r.l., Milan, Italy. Prior to the first surgery, all rats were individually housed in separate cages for a period of 7 days. The room temperature was monitored daily and maintained at 23 °C. Rats were provided with a commercial diet and had access to water ad libitum with their cages being cleaned daily.

### 5.2. Preparation of PHH

The PHHs were prepared following the previously described method [20]. Briefly, an initial solution consisting of manuka honey (Manuka health NZ Ltd. 66 Weona Court, Te Awamutu 3800, New Zealand) and sterile deionized water in a 1:1 *v*/*v* ratio was prepared. Pectin powder (Sigma-Aldrich, Milan, Italy) was gradually added (0.5:1 *w*/*v*), and the mixture was continuously stirred until it achieved homogeneity. The resulting gel was then spread into a 2 mm thick film, hot air-dried at 40 ± 0.5 °C for 6 h, cut into rectangles measuring 1.5 × 2.5 cm, and further conditioned in an air drier at 25 ± 1 °C for 5 days. Subsequently, the PHH were packed into polyethylene bags under vacuum conditions before undergoing sterilization through gamma-irradiation at 25 kg grays (kGs) (Sterigenics International LLTC, Minerbio, Bologna, Italy).

### 5.3. Bacterial Growth

Briefly, three potentially pathogenic organisms, *Staphylococcus pseudintermedius*, *Escherichia coli* and *Enterococcus faecium*, which are commonly involved in inflammatory and infective processes, were isolated from the cecal content of rats. Each isolate was identified using Matrix-assisted laser desorption ionization-time of flight mass spectrometry (MALDI-TOF MS), and were cultivated in a liquid medium (Tryptone Soy Broth, TSB, Oxoid) for 24 h at 37 °C. Subsequently, each strain was adjusted to a cell density of 1 × 10^5^ cells/mL, mixed in equal proportions, and used as inoculants.

### 5.4. First Surgery: Abdominal Defect Creation

**Figure 8:**
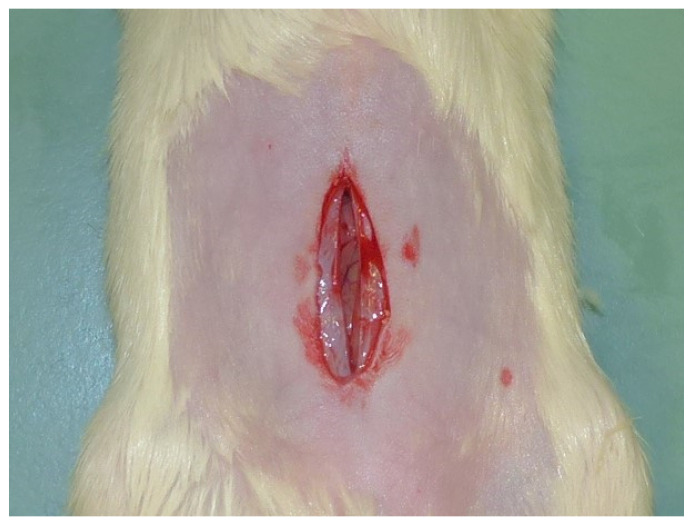
Creation of abdominal defect on skin, *linea alba* and peritoneum.

For the first surgery, rats were induced into general anesthesia through intramuscular administration of 30 mg/kg of ketamine (Lobotor^®^; Acme Srl, Corte Tegge-Cavriago, RE, Italy), 0.1 mg/kg of medetomidine (Dormisan^®^; ATI Azienda Terapeutica Veterinaria Srl, Milan, Italy), and 1 mg/kg of butorphanol (Nargesic^®^; Acme Srl, Corte Tegge-Cavriago, RE, Italy). Additionally, 5 mL isotonic sodium chloride solution (Sodio Cloruro 0.9%; Galenica Senese, Monteroni d’Arbia, Siena, Italy) was administered subcutaneously before the surgery. Each animal received 100% oxygen via a non-rebreather mask (Anesthetic face mask, XS; Jørgen Kruuse A/S, Langeskov, Denmark). A multiparameter monitoring system (Infinity Delta^®^; Dräger Italia SpA, Corsico, Italy) was used to measure heart rate through electrocardiography, peripheral oxygen saturation (SpO2) via pulse oximetry, and respiratory rate through observing chest movements. All rats were then shaved, and the surgical field was prepared using chlorhexidine digluconate and chlorhexidine alcohol solutions. A 4 cm left paramedian skin incision was created, the subcutaneous tissue was dissected, and a skin flap was raised. A full-thickness abdominal midline defect measuring 3 cm in length, including the linea alba and peritoneum was then created (Figure 8).

The defect in the linea alba was intentionally left unrepaired, while skin was closed using intradermal sutures with 5-0 USP Polyglycolic acid (Medtronic S.p.a. Milan, Italy) and skin staples. Following each surgical procedure, each rat received a subcutaneous injection of 5 mg/kg of Carprofen (Rimadyl^®^ Iniettabile; Zoetis Italia Srl, Rome, Italy) and 0.5 mg/kg of atipamezole (Sedastop^®^; Ecuphar Italia Srl, Milan, Italy) administered intramuscularly. Each rat was placed in a cage filled with oxygen until complete recovery. The rats were then housed individually with access to food and water, with daily monitoring. A period of 28 days was allocated for the maturation of the hernia defect. After this 28-day period, the rats underwent an examination to assess the development of a ventral hernia, as previously described [38].

### 5.5. Second Surgery: Abdominal Defect Repair and Contamination

**Figure 9:**
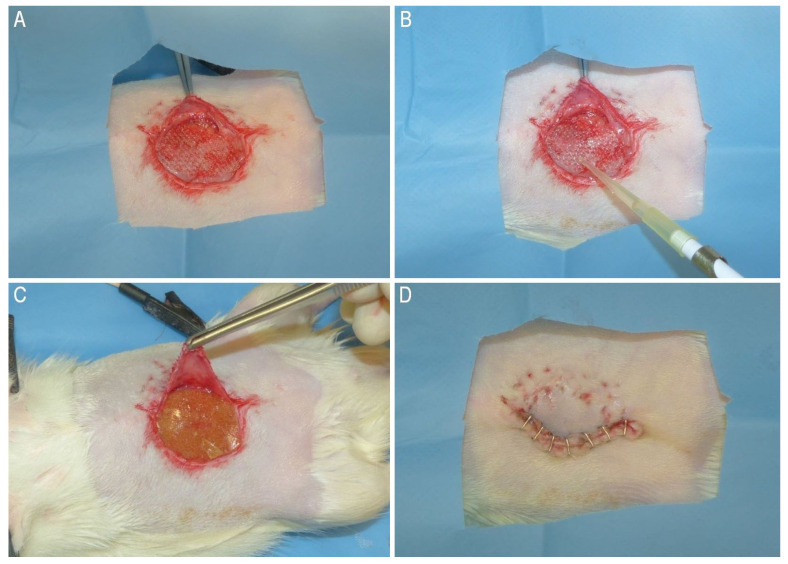
(**A**): PP mesh inserted on the linea alba to ensure the hernia repair; (**B**): contamination of the PP mesh with 0.1 mL of bacteria broth; (**C**): PHH inserted on the PP mesh before skin closure in Group T; (**D**): skin closure with skin staples.

The rats were randomly assigned to either the “treated” (T) or “control” (C) group using a free online calculator (www.random.org, accessed in September 2022). Prior to inoculation of bacteria broth, a sterile preparation of the surgical field was conducted to ensure aseptic conditions. The same anesthetic and analgesic protocol described for the previous surgery was employed. A skin incision was made to expose the hernia sac, and a 1.5 × 2.5 cm piece of mesh was applied to repair the previously created abdominal wall defect in both groups. The mesh used for repair was a PP mesh (Bard^®^ Mesh, Franklin Lakes, NJ, USA), which was inserted to cover the defect (Figure 9A). The meshes were fixed on the edges of the defect using interrupted sutures with 5-0 USP Polyglycolic acid. Subsequently, in both groups, the repair procedure was followed by the inoculation of 0.1 mL bacterial mixed culture onto the mesh implant, as previously described (Figure 9B). In the group T, a PHH was placed onto the PP mesh before closing the skin (Figure 9C). The skin was then closed using a continuous intradermal suture of 5-0 USP Polyglycolic acid and skin staples (Figure 9D).

A pain score based on the rat grimace scale [39] was assigned at 6 and 24 h after the surgery, and additional analgesics were administered as needed. All rats were housed in single cages and provided with a commercial diet, with water available ad libitum.

### 5.6. Macroscopical Evaluation

**Figure 10:**
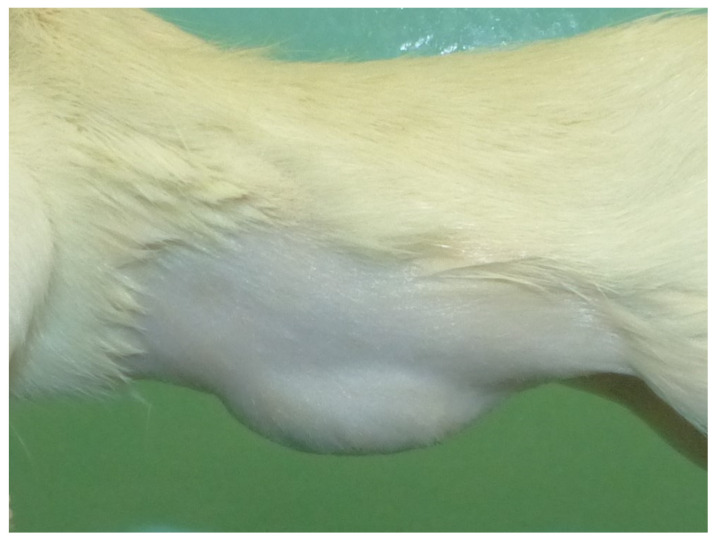
Presence of hernial defect evaluated before reparative surgery, left lateral view.

Fourteen days following the treatment procedure and the subsequent bacterial inoculation, the rats were anesthetized using the same anesthesia protocol previously described. Subsequently, cervical dislocation was performed and the presence of hernia defect was assessed in all cases (Figure 10).

The presence or absence of skin erythema and any sign of wound infection were documented. The abdomen was shaved, and a surgical scrub was performed, as previously described. A transverse incision of the wound was performed, and a sterile swab of the linea alba was collected for microbiological evaluation. Following this, a full-thickness incision around the mesh was created to assess the grade of hernia healing. To evaluate the extent of infection, a composite score referred to as the Mesh Infection Severity Index (MISI) was employed, as previously detailed in a prior study [38]. The index considered several parameters, including skin erythema, mesh adhesion severity, adhesion surface area, abscess formation, and mesh contracture.

### 5.7. Bacteriological Evaluation

The swabs were placed in a sterile diluent (buffered peptone water; Oxoid) and then vortexed. From this initial dilution, two sequential 1/100 dilutions were prepared, and 100 microliters were plated onto Tryptone Soy Agar (Oxoid, TSA) and McConkey agar (Oxoid, McC). The agar plates were incubated at 37 °C for 24 and 48 h, after which the colony count per swab was determined.

### 5.8. Histological Evaluation and Immunohistochemical Analysis

The abdominal wall was harvested and fixed in 10% formaldehyde-buffered solution before being processed for histological examination to assess the healing process. After processing, the samples were blindly evaluated by an expert pathologist (L.M.) after hematoxylin–eosin staining. A numerical score (ranging from 1 to 4) was assigned to each parameter considered, following the methodology outlined in a previous study by Pereira-Lucena and colleagues (2014) [40]. The parameters evaluated included tissue maturation, inflammatory response on mesh surface, inflammatory reaction in the host tissue and cell layers at the margins of the granulomas.

Immunohistochemistry was performed on 4 µm section of formalin-fixed, paraffin-embedded tissue using a COX-2 -rabbit polyclonal antibody (Ab15191; Cambridge, UK) diluted 1:100 and revealed as previously described [24]. The immunohistochemistry results were analyzed according to the method proposed by Pereira-Lucena and colleagues (2014) [40], utilizing a numerical score ranging from 1 to 3.

### 5.9. Statistical Analysis

Statistical analyses were carried out using commercially available software (Graph Pad, San Diego, CA, USA). Data obtained from macroscopic evaluations of hernia healing were analyzed using the Shapiro–Wilk normality test and were compared using the Mann–Whitney test. Data derived from the swab cultures were reported as either the presence (yes) or absence (no) of bacterial growth, and the two groups were compared using the Chi-square test. Data concerning the margin of granuloma inflammation induced in the host tissue via the mesh, tissue maturation, and the evaluation of COX-2 expression were analyzed using the Shapiro–Wilk normality test and compared using the Mann–Whitney test. Statistical significance was set at *p* < 0.05.

## Figures and Tables

**Table 1:** Data on CFU/mL in Group T and Group C after 24 and 48 h.

	T (*n* = 9)	C (*n* = 8)	*p* Value
CFU/mL 24 h	2	100	0.672
	2	59	
	20	0	
	15	12	
	0	0	
	0	0	
	25	0	
	1	0	
	0		
CFU/mL 48 h	2	406	0.869
	0	62	
	25	0	
	27	12	
	0	1	
	0	0	
	28	2	
	1	0	
	1		

**Table 2:** Data on histological score attributed to cases in treated and untreated group. The scores were compared through a Mann–Whitney test.

	T (*n* = 9)	C (*n* = 8)	*p* Value
	Median (range)		
Cells layers at margins of the granulomas	3 (2–3)	2,5 (2–3)	0.59
Inflammatory reaction in the host tissue	3 (2–4)	2 (2–3)	0.23
Inflammatory response on the mesh surface	3 (2–4)	3 (2–4)	0.63
Tissue maturation	3 (2–4)	2 (1–3)	0.28

**Table 3:** Data on immunohistochemistry scores to evaluate COX-2 expression.

	T (*n* = 9)	C (*n* = 8)	*p* Value
	Median (range)		
COX % cell	2 (1–3)	2 (1–3)	0.59
COX intensity	2 (1–3)	2 (1–3)	0.52

## Data Availability

The data presented in this study are contained within the article.
